# Novel Phenotype of LMNA Variant c.154C>G Affecting Heart, Liver, and Lipid and Iron Metabolism: A Case Report

**DOI:** 10.7759/cureus.38860

**Published:** 2023-05-10

**Authors:** Josef Finsterer, Gerhard Pölzl

**Affiliations:** 1 Neurology, Neurology and Neurophysiology Center, Vienna, AUT; 2 Internal Medicine, Medical University of Innsbruck, Innsbruck, AUT

**Keywords:** skeletal muscle, laminopathy, heart failure, lamin a/c, dilative cardiomyopathy

## Abstract

Mutations in the *LMNA* gene cause heterogeneous phenotypes such as myopathy, progeroid syndromes, hereditary neuropathies, cardiomyopathies, or lipodystrophies. A specific *LMNA* mutation manifesting as dilated cardiomyopathy (dCMP), and iron metabolism disorder has not been reported. The patient is a 50-year-old female with palpitations and fatigue since childhood, hyperlipidemia for 25 years, gastroesophageal reflux for 20 years, arterial hypertension for eight years, and iron deficiency for one year, requiring intravenous iron supplementation. Family history was positive for dCMP, malignant ventricular arrhythmias (MVAs), and sudden cardiac death (SCD). She was diagnosed with dCMP at the age of 49. Genetic workup revealed the variant c.154C>G (p.Leu52Val) in *LMNA*, which was also found in two female cousins. Because of ventricular tachycardia in the long-term ECG recordings, an implantable cardioverter-defibrillator (ICD) was implanted in addition to antiarrhythmic, antihypertensive, heart failure, and lipid-lowering treatment. With this therapy, the patient remained in stable condition during the one-year follow-up and was able to successfully carry out her job. In summary, this case shows that the variant c.154C>G (p.Leu52Val) in *LMNA* manifests not only with dCMP, but also with hyperlipidemia, steatosis, gastroesophageal reflux, arterial hypertension, and iron deficiency. Primary prophylaxis with an ICD and additional symptomatic treatment can stabilise the condition and eventually prevent familial SCD.

## Introduction

The lamin A/C gene (*LMNA*) encodes several slightly different proteins known as lamins [1]. The two main proteins produced from the gene in most of the cells are lamin A and lamin C [1]. Lamins A/C are intermediate filament proteins that provide stability and strength to cells as they act as scaffolding components of the nuclear envelope [1]. Mutations in this gene cause heterogeneous phenotypes, such as myopathy (e.g. Emery-Dreifuss muscular dystrophy (ED-MD)), progeroid syndromes (e.g. Hutchinson-Gilford progeria syndrome, Werner syndrome), hereditary neuropathies, cardiomyopathies (e.g. dilated cardiomyopathy (dCMP)), or lipodystrophies [2,3]. This case report describes a patient with laminopathy due to a rare *LMNA* variant phenotypically manifesting in the heart, lipid metabolism, and iron metabolism.

## Case presentation

**Figure 1:**
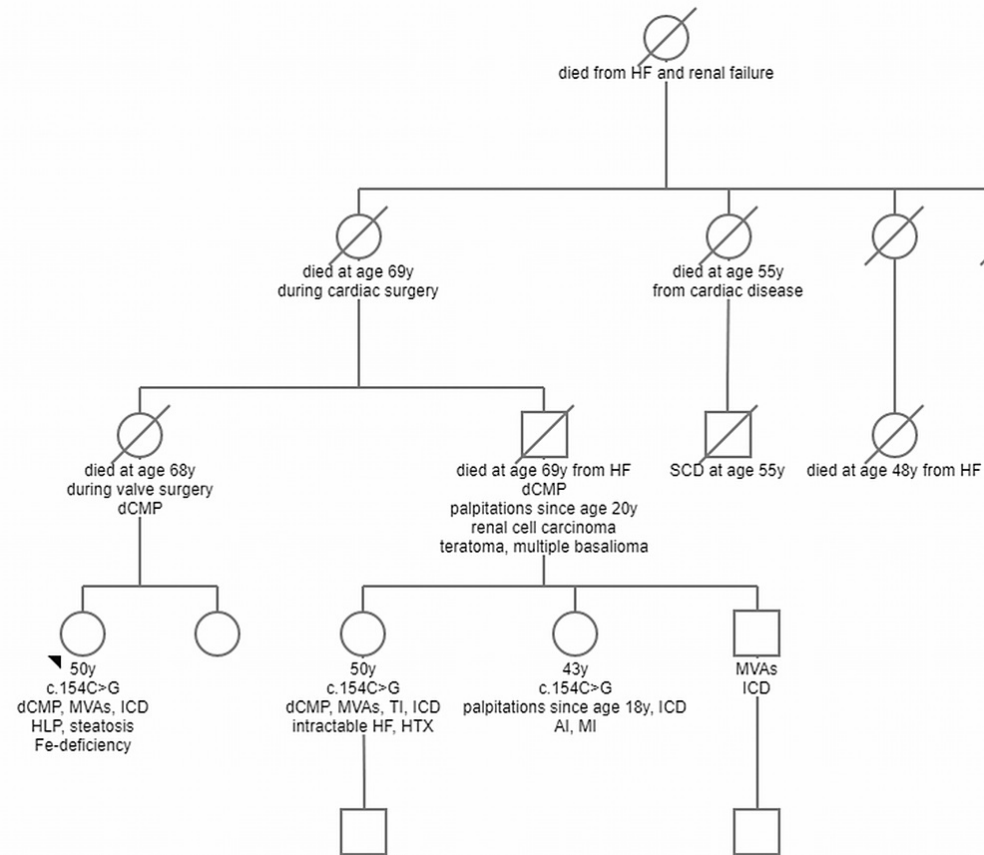
Pedigree of the index patient AI: aortic insufficiency, dCMP: dilated cardiomyopathy, HF: heart failure, HLP: hyperlipidemia, ICD: implantable cardioverter defibrillator, MI: mitral insufficiency, MVA: malignant ventricular arrhythmia, SCD: sudden cardiac death, TI: tricuspid insufficiency

The patient is a non-smoking and non-alcoholic 50-year-old Caucasian female, height 162 cm, weight 60 kg, who was diagnosed with dCMP at the age of 49 years. Genetic processing revealed the *LMNA* variant c.154C>G (p.Leu52Val) to be the cause. The mutation had previously been identified in two of her female cousins (Figure 1). Her history was positive for palpitations and recurrent fatigue since childhood, hyperlipidemia for 25 years, treated with statins for two years, gastro-oesophagal reflux for 20 years, arterial hypertension for eight years, hepatic steatosis for one year, iron deficiency since age 50 requiring intravenous iron substitution, hypermetropia, and allergy to wasps and sulfonamides.

The family history was positive for heart disease in several first-degree relatives (Figure 1). Two female cousins also carried the c.154C>G variant. Both were implanted with an implantable cardioverter defibrillator (ICD) and the older of the two even required heart transplantation (HTX) for treatment-resistant heart failure (Figure 1). The brother of these two cousins also received an ICD but refused to undergo a genetic test (Figure 1). The father of these three siblings died from heart failure at the age of 69 despite having an ICD implantation. The index patient’s grandmother died during cardiac surgery at the age of 69 (Figure 1). Her sisters died of heart failure at ages 55 and 48, respectively. The index patient’s great-grandmother died of heart failure and renal insufficiency (Figure 1).

**Figure 2:**
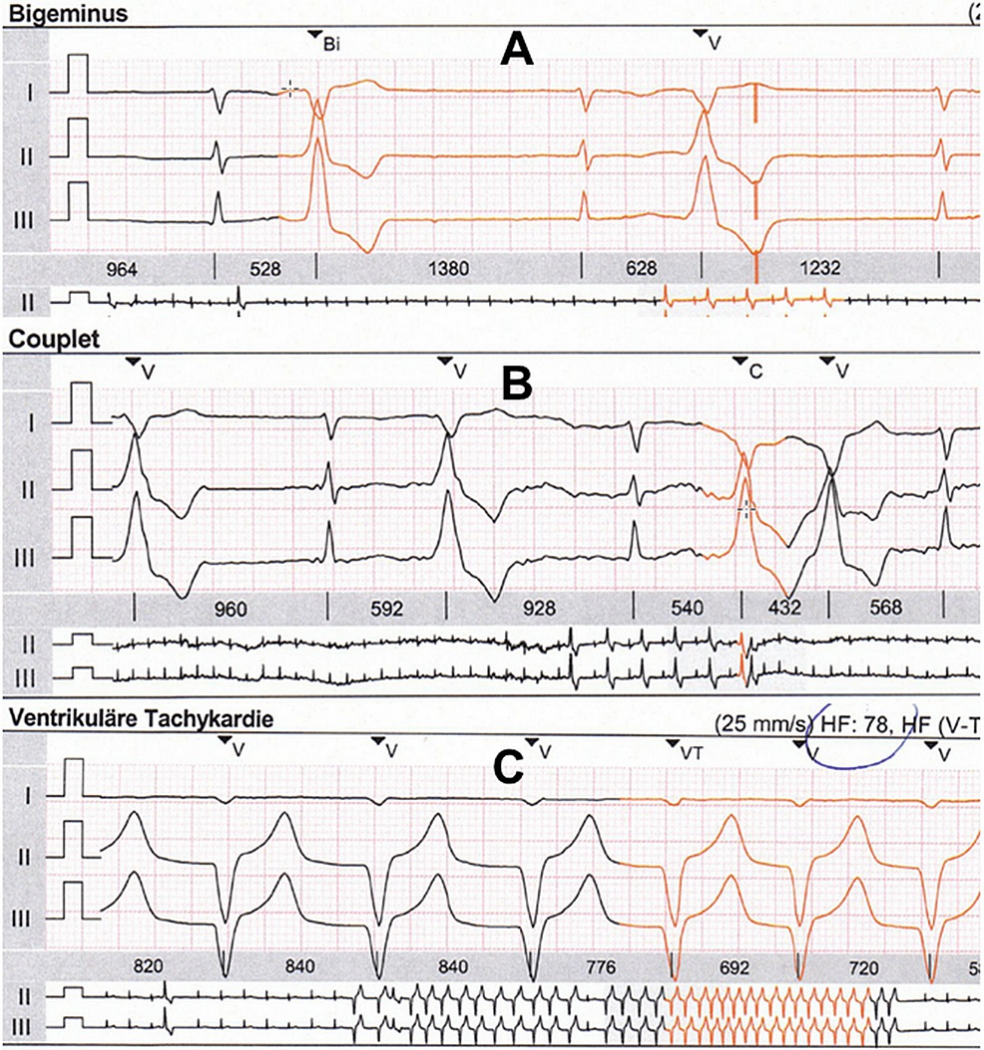
Ventricular arrhythmias, including bigeminus (panel A), couplets (panel B), and spontaneous ventricular tachycardia of 26 seconds duration (panel C) were the primary indication for implantation of an ICD ICD: implantable cardioverter-defibrillator

**Figure 3:**
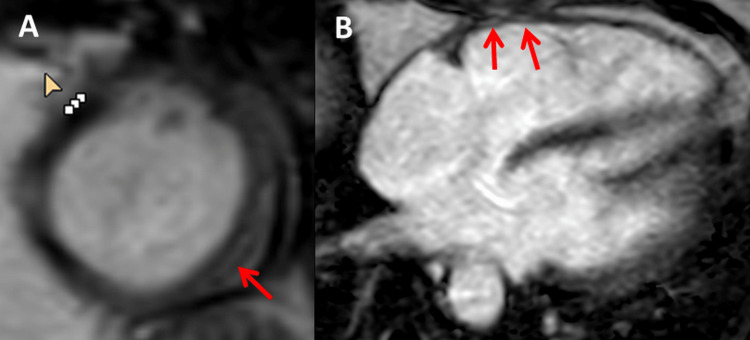
Cardiac MRI at age 49 years showing subepicardial LGE 4-12 minutes after intravenous application of gadolinium in the lateral and posterior wall of the left ventricular myocardium on 3D-T1-TFE sequences (panel A) and on T1w-PSIR sequences (panel B) LGE: late gadolinium enhancement; 3D: three dimensional; TFE: turbo field echo; PSIR: phase-sensitive inversion recovery

At the age of 48, the index patient developed exercise-induced dizziness, visual disturbances, dyspnoea, loss of performance, general weakness, and a feeling of fainting, which was attributed to arterial hypertension. Her condition improved with new antihypertensive drugs without returning to pre-morbid levels. A Holter-ECG monitoring at age 49 showed paroxysmal atrial fibrillation, frequent ventricular ectopic beats, intermittent left bundle branch block, ventricular bigeminy, and a non-sustained ventricular tachycardia lasting 26 seconds (Figure 2). Echocardiography showed reduced systolic function, severe tricuspid regurgitation 3+, moderate mitral regurgitation 2+, and moderate aortic regurgitation 2+. N-terminal pro-brain natriuretic peptide (NT-proBNP) was 1000 ng/l (n, <125 ng/l). Heart failure treatment was started. An abdominal ultrasound showed hepatic steatosis. A cardiac MRI at age 49 showed mild to moderate mitral regurgitation, mild diffuse hypokinesia of the lateral, left ventricular wall, mild fatty infiltration of the right ventricular wall, and band-like, subepicardial late gadolinium enhancement (LGE) in the left ventricular lateral and posterior wall (Figure 3). Coronary angiography showed no significant coronary artery disease (CAD). Because of the malignant ventricular arrhythmias (MVAs) on Holter monitoring, an ICD (VIGILANT^TM^; Boston Scientific Corporation, Marlborough, Massachusetts, United States) was implanted as primary prophylaxis. She did not undergo an electrophysiological study (EPS) or pulmonary vein isolation (PVI) prior to implantation of the ICD because MVAs were already identified on Holter monitoring. Due to the intermittent left bundle branch block, there was no indication for the implantation of a cardiac resynchronization therapy (CRT) system. Follow-up echocardiography showed systolic function in the lower normal range (ejection fraction (EF) 53%), moderate tricuspid regurgitation 2+, mitral regurgitation 2+, and mild aortic regurgitation 1+. Follow-up NT-proBNP was 544 ng/l.

One year after implantation of the ICD, the patient was referred for questionable skeletal muscle involvement. The patient was able to climb two flights of stairs without any problems and walk independently on level ground. She was able to do her job without problems. She did not report any symptoms suggestive of myopathy. Family history was negative for neuromuscular disorders. The ICD has never discharged since implantation. Clinical neurologic exam only revealed hypermetropia and weak tendon reflexes. The patient reported orthostatic dizziness when rising from a lying position. Blood tests revealed mild anemia, moderate renal insufficiency (glomerular filtration rate (GFR): 56 ml/min), prediabetes (glycated hemoglobin (HbA1c) 6.3), reduced transferrin saturation (9%), elevated transferrin (381 mg/dl), increased total iron binding (537 mg/dl), and hypertriglyceridemia. Muscle enzymes (creatine-kinase (CK), lactate-dehydrogenase (LDH), aldolase, and myoglobin) and lactate were within the normal range. Workup for iron deficiency (hemoglobinopathies, coeliac screen, gastroscopy, colonoscopy, screening for intoxication, urine microscopy) was non-informative. Needle electromyography of the anterior tibial muscle was normal and nerve conduction studies excluded polyneuropathy. Cerebral computed tomography showed only calcifications of the falx. Evaluation for renal disease was non-informative. Her latest medication included spironolactone (50 mg/d), bisoprolol (5 mg/d), edoxaban (60 mg/d), rosuvastatin (10 mg/d), esomeprazole (40 mg/d), escitalopram (10 mg/d), torasemide (10 mg/d), sacubitril/valsartan (97/103 mg/d), and dapagliflozin (10 mg/d).

## Discussion

The presented patient is interesting for a novel phenotype of the *LMNA* variant c.154C>G, which manifested not only with dCMP and MVAs, but also with hepatic steatosis, iron deficiency, hyperlipidemia, gastroesophageal reflux, and arterial hypertension. Whether these additional features were really due to the mutation or were accidental remains speculative. The mutation did not manifest with myopathy, lipodystrophy, progeria, or hereditary neuropathy, common manifestations of *LMNA* variants. The most common phenotypic manifestations of *LMNA* mutations are cardiomyopathy and myopathy. Several patients with dCMP due to different *LMNA* mutations have been reported [4,5]. The first report on *LMNA*-associated dCMP was published in 1999 [6]. The variant c.154C>G has so far only been described in a single patient [7]. This patient presented only with dCMP [7]. dCMP due to *LMNA* mutations can be complicated by supraventricular arrhythmias and MVAs or heart failure and requires prophylactic treatment with an ICD or CRT and, in case of heart failure, treatment with heart failure therapy. When heart failure becomes intractable, heart transplantation is an option, as in the index patient’s cousin and several patients in the literature [8].

Late gadolinium enhancement (LGE) has only rarely been reported in LMNA mutation carriers [9,10]. In a recent study of 42 patients with laminopathy, cardiac MRI showed LGE in 61% [9]. Of the entire cohort, 56% of patients required implantation of an ICD [9]. Of these, 20% developed MVAs during a mean follow-up of 10 years. No difference regarding the risk factors for sudden cardiac death was found between LGE-positive and LGE-negative patients with laminopathy [9]. However, it was concluded that the presence of LGE is significantly associated with the occurrence of MVAs [9]. The absence of LGE allowed the exclusion of MVAs during a 10-year follow-up [5]. Because the history was negative for biopsy-proven myocarditis, LGE in the index patient was interpreted as myocardial fibrosis due to laminopathy rather than a residual lesion after myocarditis.

Whether iron deficiency was causally related to the *LMNA* variant or accidental remains speculative. Alternative causes of iron deficiency were excluded. Iron deficiency has not previously been described as a manifestation of *LMNA* mutations. The pathophysiology of iron deficiency, reduced transferrin saturation, increased transferrin, and increased total iron binding due to *LMNA* mutations remains unclear for the time being, but it can be speculated that the variant c.154C>G also affected iron transport and metabolism within cells or mitochondria. The only indication for a disturbed iron metabolism by *LMNA* variants is a mouse model of the Hutchinson-Gilford progeria syndrome, which mainly manifests itself in atherosclerosis, but also in premature aging, reduced body weight, and reduced survival time [11]. Mouse aortas showed vascular smooth muscle cell depletion in the media, adventitial thickening, and changes in elastin structure [11]. Atheromas of Ldlr-/-LmnaG609G/G609G mice exhibited features of unstable plaques, including the presence of erythrocytes and iron deposits, and reduced levels of smooth muscle cells and collagen [11]. No premature atherosclerosis with iron deposits was found in the index patient. Iron deficiency in the index patient could not be explained by reduced intake because she ate a normal diet and had no impaired duodenal absorption, no increased iron deposition, and no increased iron loss via the intestines or kidneys. However, it is conceivable that iron storage decreased due reduced availability of iron transport proteins.

Hyperlipidemia or dyslipidemia has also been reported in carriers of *LMNA* mutations [12,13]. Hyperlipidemia can secondarily cause steatosis. Hepatic steatosis has been particularly reported in *LMNA* mutation carriers manifesting with familial lipodystrophy [14]. Arterial hypertension has also been reported occasionally in carriers of *LMNA* mutations [15,16].

## Conclusions

This case shows that the variant c.154C>G (p.Leu52Val) in LMNA can manifest not only with dCMP but also with arterial hypertension, hyperlipidemia, hepatic steatosis, reflux disease, and iron deficiency. Primary prophylaxis of SCD due to MVAs with an ICD and additional symptomatic treatment can lead to a stable condition of affected patients and optimal prevention of familial SCD due to MVAs.

## References

[1] Hershberger RE, Jordan E (2022). LMNA-related dilated cardiomyopathy. GeneReviews® [Internet].

[2] Ho R, Hegele RA (2019). Complex effects of laminopathy mutations on nuclear structure and function. Clin Genet.

[3] He G, Yan Z, Sun L, Lv Y, Guo W, Gang X, Wang G (2019). Diabetes mellitus coexisted with progeria: a case report of atypical Werner syndrome with novel LMNA mutations and literature review. Endocr J.

[4] Perrot A, Hussein S, Ruppert V (2009). Identification of mutational hot spots in LMNA encoding lamin A/C in patients with familial dilated cardiomyopathy. Basic Res Cardiol.

[5] Parks SB, Kushner JD, Nauman D (2008). Lamin A/C mutation analysis in a cohort of 324 unrelated patients with idiopathic or familial dilated cardiomyopathy. Am Heart J.

[6] Fatkin D, MacRae C, Sasaki T (1999). Missense mutations in the rod domain of the lamin A/C gene as causes of dilated cardiomyopathy and conduction-system disease. N Engl J Med.

[7] Lakdawala NK, Funke BH, Baxter S (2012). Genetic testing for dilated cardiomyopathy in clinical practice. J Card Fail.

[8] Saj M, Bilinska ZT, Tarnowska A (2013). LMNA mutations in Polish patients with dilated cardiomyopathy: prevalence, clinical characteristics, and in vitro studies. BMC Med Genet.

[9] Peretto G, Barison A, Forleo C (2020). Late gadolinium enhancement role in arrhythmic risk stratification of patients with LMNA cardiomyopathy: results from a long-term follow-up multicentre study. Europace.

[10] Wang S, Peng D (2020). Case series: LMNA-related dilated cardiomyopathy presents with reginal wall akinesis and transmural late gadolinium enhancement. ESC Heart Fail.

[11] Nevado RM, Hamczyk MR, Gonzalo P, Andrés-Manzano MJ, Andrés V (2020). Premature vascular aging with features of plaque vulnerability in an atheroprone mouse model of Hutchinson-Gilford progeria syndrome with ldlr deficiency. Cells.

[12] Hegele RA, Kraw ME, Ban MR, Miskie BA, Huff MW, Cao H (2003). Elevated serum C-reactive protein and free fatty acids among nondiabetic carriers of missense mutations in the gene encoding lamin A/C (LMNA) with partial lipodystrophy. Arterioscler Thromb Vasc Biol.

[13] Fernandez-Pombo A, Diaz-Lopez EJ, Castro AI, Sanchez-Iglesias S, Cobelo-Gomez S, Prado-Moraña T, Araujo-Vilar D (2023). Clinical spectrum of LMNA-associated type 2 familial partial lipodystrophy: a systematic review. Cells.

[14] Treiber G, Flaus Furmaniuk A, Guilleux A (2021). A recurrent familial partial lipodystrophy due to a monoallelic or biallelic LMNA founder variant highlights the multifaceted cardiac manifestations of metabolic laminopathies. Eur J Endocrinol.

[15] Nakajima K, Aiba T, Makiyama T (2018). Clinical manifestations and long-term mortality in lamin A/C mutation carriers from a Japanese multicenter registry. Circ J.

[16] Patni N, Xing C, Agarwal AK, Garg A (2017). Juvenile-onset generalized lipodystrophy due to a novel heterozygous missense LMNA mutation affecting lamin C. Am J Med Genet A.

